# Predicting multi-vascular diseases in patients with coronary artery disease

**DOI:** 10.12688/f1000research.134648.1

**Published:** 2023-06-26

**Authors:** Suko Adiarto, Luthfian Aby Nurachman, Raditya Dewangga, Suci Indriani, Taofan Taofan, Amir Aziz Alkatiri, Doni Firman, Anwar Santoso

**Affiliations:** 1 Department of Cardiology and Vascular Medicine, Faculty of Medicine, Universitas Indonesia, National Cardiovascular Center Harapan Kita, Jakarta, Indonesia; 2 Faculty of Medicine, Universitas Indonesia, Depok, West Java, Indonesia; 3 Gunung Jati General Hospital, Cirebon, West Java, Indonesia

**Keywords:** Coronary artery disease, Peripheral artery disease, Abdominal Aortic Aneurysm, Carotid Artery Stenosis, targeted screening, predictive model, clinical risk score

## Abstract

**Background:** Because of its systemic nature, the occurrence of atherosclerosis in the coronary arteries can also indicate a risk for other vascular diseases.  However, screening program targeted for all patients with coronary artery disease (CAD) is highly ineffective and no studies have assessed the risk factors for developing multi-vascular diseases in general. This study constructed a predictive model and scoring system to enable targeted screening for multi-vascular diseases in CAD patients.

**Methods**: This cross-sectional study includes patients with CAD, as diagnosed during coronary angiography or percutaneous coronary intervention from March 2021 to December 2021. Coronary artery stenosis (CAS) and abdominal aortic aneurysm (AAA) were diagnosed using Doppler ultrasound while peripheral artery disease (PAD) was diagnosed based on ABI score. Multivariate logistic regression was conducted to construct the predictive model and risk scores. Validation was conducted using ROC analysis and Hosmer-Lemeshow test.

**Results**: Multivariate analysis showed that ages of >60 years (OR [95% CI] = 1.579 [1.153-2.164]), diabetes mellitus (OR = 1.412 [1.036-1.924]), cerebrovascular disease (OR = 3.656 [2.326-5.747]), and CAD3VD (OR = 1.960 [1.250-3.073]) increased the odds for multi-vascular disease. The model demonstrated good predictive capability (AUC = 0.659) and was well-calibrated (Hosmer-Lemeshow p = 0.379). Targeted screening for high-risk patients reduced the number needed to screen (NNS) from 6 in the general population to 3 and has a high specificity of 96.5%

**Conclusions:** Targeted screening using clinical risk scores was able to decrease NNS with good predictive capability and high specificity

## Introduction

Atherosclerosis underlines some of the major cardiovascular diseases which are the leading cause of mortality worldwide. Due to its systemic nature, atherosclerosis in the coronary arteries may indicate occurrences in other arteries. ^1^ Coronary arterial disease (CAD) is associated with peripheral artery disease (PAD), carotid artery stenosis, and abdominal aortic aneurysm (AAA). There is an increasing prevalence of vascular diseases in patients with a more severe CAD, especially high-risk CAD with metabolic syndromes and three-vessel disease. ^2^^–^^4^ Therefore, the screening in patients with CAD is justifiable in assessing the risk of atherosclerosis occurrences in other arteries.

CAD is one of the leading causes of mortality worldwide with a substantial incidence rate. According to the Centers for Disease Control and Prevention (CDC) in the United States, a total of 20.1 million adults aged 20 years old and older have CAD. ^5^ Screening for PAD, carotid artery stenosis, and AAA enable early detection, risk stratification, and early cardiovascular treatment; all in favor of reducing morbidity and mortality. ^6^^,^^7^ However, screening program targeting all CAD patients is highly ineffective, costly, and requires multiple resources such as specific instruments and trained technicians. On the other hand, screening for early detection of some vascular diseases, e.g., PAD, in patients with significant CAD was not shown to be more beneficial compared to a routine medical checkup. ^8^ Therefore, targeted screening in patients with CAD requires careful consideration based on risk factors for atherosclerosis in other arteries.

Targeted screening contributes to early detection while being more time and cost-effective than general screening; it can be beneficial for diseases previously deemed not necessary to screen. A study specifically assessed targeted screening for AAA was already done involving a small subset of indigenous people in Borneo. ^9^ No research has been conducted to assess the predictors for developing multi-vascular diseases, including PAD, CAS, and AAA in patients with CAD. Furthermore, no studies assessed the number needed to screen (NNS) of screening for multi-vascular diseases in CAD patients and the impact of the clinical risk scoring system for said NNS. We hypothesize that a risk-scoring tool to measure the risk of other vascular diseases in CAD patients is feasible to construct and can be applied for targeted screening; in return, the risk-scoring tool can reduce the NNS for asymptomatic multi-vascular diseases. Therefore, this study aims to investigate factors predicting the occurrence of multi-vascular diseases in patients with CAD while constructing a predictive model and scoring system to enable targeted screening for future uses.

## Methods

This study was conducted based on the STROBE guideline for observational studies. The study protocol was approved by the National Cardiovascular Center Harapan Kita Hospital committee of ethics (ethics approval number: LB.02.01/VII/509/KEP005/2021). All patients gave written informed consent prior to the recruitment of the study and were free to decline participation.

### Patient selection

This cross-sectional study was conducted at the National Cardiovascular Center of Harapan Kita Hospital from March 2021 to December 2021. All patients who underwent elective coronary angiography or percutaneous coronary intervention from March 2021 to December 2021 were initially included. Patients diagnosed with coronary artery disease (CAD) were eligible for inclusion in our study. We excluded patients with previously diagnosed CAD that were treated by medical therapy and revascularization therapy. We also excluded patients with connective tissue disorder. All included patients were concomitantly screened for vascular disease. The primary outcome of interest was vascular diseases in other vascular territories. For all patients, we examined the list of variables relating to sociodemographic characteristics, cardiovascular risk factors, and other related diseases.

The diagnosis of hypertension was based on documented medical history, the use of antihypertensive drugs, and the presence of elevated systolic and/or diastolic blood pressure according to European Society of Cardiology guidelines. ^10^ Diabetes mellitus was diagnosed based on documented medical history through the use of hypoglycemic agents, and/or laboratory criteria according to the American Diabetes Association (ADA) 2021. ^11^ Dyslipidemia was defined based on documented medical history and the use of lowering lipid agents or laboratory criteria according to the National Cholesterol Education Program’s Adult Treatment Panel III (NCEP-ATP III). ^12^ Metabolic Syndrome was also defined according to the NCEP-ATP III. Information on the history of cerebrovascular disease (transient ischemic attack or stroke) was collected from the patient’s reports or medical records. ^12^ CAD was diagnosed using angiography. The presence of coronary lesions was determined using visual estimation. Coronary artery lesions were considered as CAD if 1) at least one major epicardial artery or its major branches have significant stenosis (70% for left anterior descending artery, left circumflex artery, right coronary artery, or 50% for left main trunk) or 2) the patient was previously hospitalied for treatment of coronary artery lesions (balloon, stent, or coronary artery bypass grafting).

Lower extremity peripheral arterial disease (PAD) was defined with 1) ankle-brachial index of < 0.9 or 2) the patient was previously treated for PAD. ^13^ Evaluation of carotid artery stenosis (CAS) and AAA was conducted using bedside ultrasound Affiniti 70 (Philips, Amsterdam, Netherlands) by a cardiovascular technician blinded to other data. Peak systolic velocity, end-diastolic velocity, and intima-media thickness of the common carotid artery and internal carotid artery were calculated to evaluate CAS. The degree of CAS was classified according to Grant *et al.* ^14^ CAS was considered significant if 1) the presence of stenosis ≥50% from ultrasound examination or 2) the patient was previously treated for CAS (carotid stenting or carotid endarterectomy). AAA was defined as an enlargement of the abdominal aorta with a diameter of ≥3 cm or a previously treated AAA lesion (Endovascular aortic repair/EVAR or open surgical repair). ^15^ The maximum and minimum abdominal aortic diameter (anteroposterior or transverse axis) were obtained.

Potential bias for each diagnosis of vascular disease were minimized by involving third-party examiners who were not aware of the existence of this study.

### Statistical analysis

Categorical variables were expressed in the form of numbers and percentages, whereas continuous variables were expressed as their mean value±SD in data with normal distribution or their median (interquartile range) value in data without normal distribution. We compared categorical variables using Pearson’s chi-square or Fisher’s exact test while continuous variables were compared using the Student t-test or Mann-Whitney U test. We conducted multivariable stepwise logistic regression to generate prediction models with the primary endpoint of multivascular disease incorporating clinical variables. All variables with *p* value of <0.25 by univariate analysis were included in the multivariable model. The selection of variables for retention was based on p value of <0.05. We additionally performed the Hosmer-Lemeshow test to assess the goodness of fit of the model and plot the observed versus predicted data graph.

For each significant variable from multivariate analysis, a regression β coefficient was obtained, and a scoring system was created to predict the incidence of coexisting vascular diseases. Points for the scoring prediction rule were assigned by weighing each significant variable compared to the total β coefficient. Then, points were made using the weighted coefficients with rounding to the nearest whole number. We created the cutoff points to classify patients with low, moderate, and high-risk probability, respectively. To test the model discrimination, C-statistic was also conducted to calculate the area under the curve. We also conducted internal validation by bootstrap using the same amount of included samples. All statistical analyses were performed using SPSS version 23 (IBM, New York, USA) and STATA version 16 MP (StataCorp, Texas, USA).

## Results

**Figure 1.**
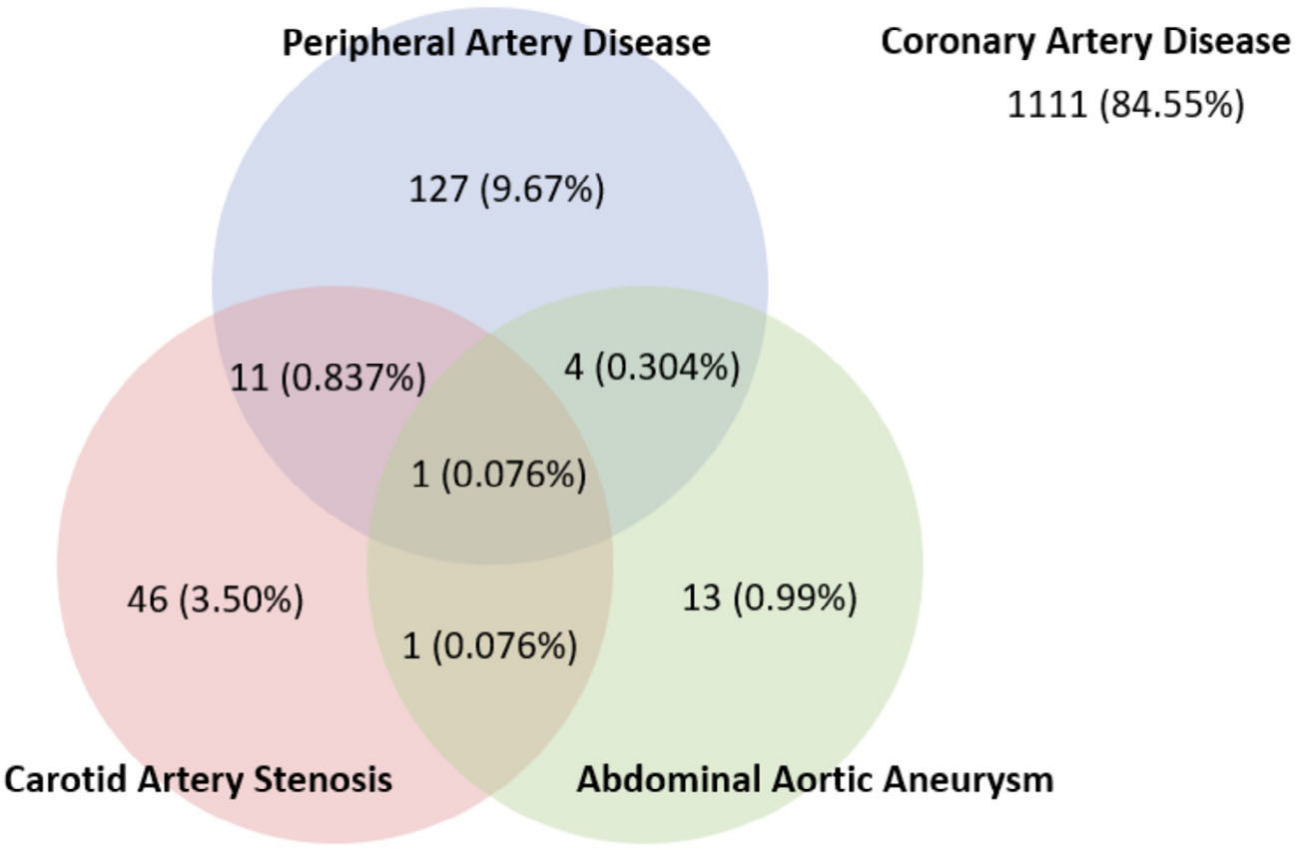
Vascular disease prevalence in CAD.

A total of 1314 patients with CAD were identified; 203 (15.4%) patients have vascular disease. All patients had complete medical record data and, therefore, no missing data in this study. Sociodemographic and clinical data are shown in Table 1. Amongst the variables in patient’s demographics, there was a significant difference in the proportion of patients with cerebrovascular disease, CAD three-vessel disease (CAD3VD), and CAD left main disease (CAD-LM). The prevalence of PAD, CAS, and AAA in patients with CAD were 143 (10.9%), 59 (4.5%), and 19 (1.4%), respectively. The overlap between vascular diseases is shown in Figure 1.

**Table 1.** Patient’s baseline characteristics. ^31^

Variable	Total (n=1314)	Multivasc Dis (+) (n=203)	Multivasc Dis (-) (n=1111)	*p* value
Age, median (IQR)	60 (13)	63 (11)	59 (13)	<0.001
Male, n (%)	1077 (82)	158 (77.8)	919 (82.7)	0.096
Obesity, n (%)	193 (14.7)	29 (14.3)	164 (85.0)	0.860
Hypertension, n (%)	1142 (86.9)	179 (88.2)	963 (86.7)	0.560
Smoker, n (%)	342 (26)	53 (26.1)	289 (26)	0.734
Ex-smoker, n (%)	541 (41.2)	87 (42.9)	454 (40.9)	0.530
Dyslipidemia, n (%)	888 (67.6)	133 (65.5)	755 (68)	0.495
Diabetes Mellitus, n (%)	583 (44.4)	107 (52.7)	476 (42.8)	0.009
Metabolic Syndrome, n (%)	671 (51.1)	102 (50.2)	569 (51.2)	0.800
Cerebrovascular Disease, n (%)	96 (7.3)	37 (18.2)	59 (5.3)	<0.001
Coronary Artery Disease 1VD, n (%)	265 (20.2)	27 (13.3)	238 (21.4)	-
Coronary Artery Disease 2VD, n (%)	392 (29.8)	41 (20.2)	351 (31.6)	0.911
Coronary Artery Disease 3VD, n (%)	657 (50)	135 (66.5)	522 (47)	<0.001
CAD LM Disease, n (%)	222 (16.9)	47 (23.2)	175 (15.8)	0.010

(+) and (-) indicates positive and negative for each disease; Multivasc Dis: multivascular disease; IQR: interquartile range; VD: vascular disease; CAD LM: coronary artery disease left main.

There is a difference in prevalence of vascular diseases in CAD patients with one, two, and three-vessel disease ( Table 2).

**Table 2.** Difference in prevalence of vascular disease between different severity of CAD. ^31^

	CAD1VD	CAD2VD	CAD3VD	*p* value
multivascular disease, n (%)				
(+)	27 (10.8)	41 (10.4)	135 (20.5)	0.000
(-)	238 (89.8)	351 (89.5)	522 (79.5)	
PAD, n (%)				
(+)	24 (9.1)	28 (7.1)	91 (13.8)	0.002
(-)	241 (90.9)	364 (92.9)	566 (86.2)	
CAS, n (%)				
(+)	2 (0.75)	7 (1.78)	50 (7.6%)	0.000
(-)	263 (99.25)	385 (98.2)	607 (92.4%)	
AAA, n (%)				
(+)	1 (0.4)	7 (1.8)	11 (1.7)	0.201
(-)	264 (99.6)	385 (98.2)	646 (98.3)	

(+) and (-) indicates positive and negative for each disease; CAD1VD: CAD one-vessel disease; CAD2VD: CAD two-vessel disease; CAD3VD: CAD three-vessel disease.

Univariate analysis demonstrated individuals with older age, diabetes mellitus, cerebrovascular disease, CAD3VD, and left main disease were more likely to have multivascular disease ( Table 3). After a multivariate analysis, four variables were retained to form the final clinical model: ages of ≥60 years (OR: 1.579; 95% CI: 1.153-2.164), diabetes mellitus (OR: 1.412; 95% CI: 1.036-1.924), cerebrovascular disease (OR: 3.656; 95% CI: 2.326-5.747), and CAD3VD (OR: 1.960; 95% CI: 1.250-3.073). All of the predictors remain significant after bootstrap internal validation ( Table 3).

**Table 3.** Factors significantly associated with multivascular disease in univariate analysis and stepwise logistic regression analysis. ^31^

Variable	Univariate Analysis		Multivariate Regression		
	OR (95% CI)	*p* value	β-coefficient	OR (95% CI)	*p* value
Age ≥ 60	1.713 (1.261-2.327)	0.001	0.457	1.579 (1.153-2.164)	0.004
Male	0.734 (0.509-1.058)	0.097	-	-	-
Diabetes Mellitus	1.487 (1.101-2.007)	0.010	0.345	1.412 (1.036-1.924)	0.029
Cerebrovascular Disease	3.974 (2.553-6.186)	<0.001	1.296	3.656 (2.326-5.747)	<0.001
CAD3VD	2.280 (1.467-3.542)	<0.001	0.673	1.960 (1.250-3.073)	0.003
Left Main Disease	1.611 (1.120-2.319)	0.01	-	-	-

OR: odds ratio; 95% CI: 95% confidence interval; CAD3VD: coronary artery disease three vessels disease.

**Figure 2.**
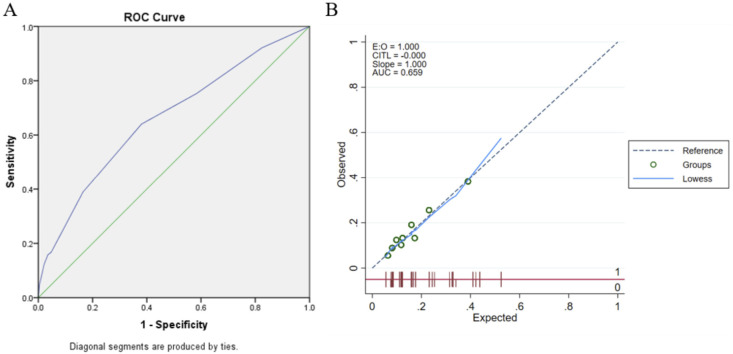
Logistic regression model’s validation (A) Discrimination using *Receiver-operator Curve* (ROC) analysis; (B) Calibration plot analysis.

The area under the curve of the model was 0.659 (95% CI: 0.617-0.700) and was well calibrated (Hosmer-Lemeshow test *p*=0.379;). Using bootstrap validation, the optimism-corrected area under the curve was 0.653 (95% CI: 0.610-0.695), which represents the predictive ability of the model ( Figure 2).

Those four variables were assigned weighted points scores for the final clinical prediction of multi-vascular disease based on the magnitude of the β coefficient ( Table 4). We designated a score of 0–2 as low probability (9% of chance), 3–5 as moderate probability (21%), and 6–8 as high probability (42%). The discriminatory ability of the scoring system was moderate (AUC: 0.649; 95% CI: 0.610-0.687).

**Table 4.** Scoring system.

Variable	Score
Age ≥ 60	1 point
Diabetes Mellitus	1 point
Cerebrovascular Disease	4 points
Coronary Artery Disease 3 Vessel Disease	2 points

Low risk 0-2, moderate risk 3-5, high risk 6-8.

**Table 5.** Performance of risk score in detecting vascular disease in CAD patients. ^31^

	Total patients (n)	Patients with multivascular disease (n)	NNS	PPV (%)	NPV (%)	Se (%)	Sp (%)
Targeted screening in all patients	1314	203	6	15.5	-	-	-
Targeted screening of those with at least moderate risk	553	130	5	23.5	90.4	64.0	61.9
Targeted screening of those with at least high risk	71	32	3	45.1	86.2	15.8	96.5

Based on our data, targeted screening for patients with moderate risk or higher decreases the number needed to screen (NNS) from six to five while targeted screening for patients with high risk reduces the NNS from six to three. Targeted screening for patients with moderate risk or higher had the most balanced results of diagnostic values with positive predictive value (PPV), negative predictive value (NPV), sensitivity, and specificity of 45,1%, 86,2%, 15,8%, and 96,5% respectively.

## Discussion

Our study reported factors associated with multi-vascular disease in patients with CAD. A total of three vascular diseases were identified in this study: PAD, CAS, and AAA. In this study, the prevalence of PAD, CAS, and AAA in patients with CAD were 143 (10.9%), 59 (4.5%), and 19 (1.4%), respectively. These numbers seem to be lower than other studies conducted elsewhere. For PAD, a study by Poredos *et al* (2007), exemplified that a total of 42% of patients with CAD had PAD based on screening using the ankle-brachial index (ABI). ^2^ For CAS, a study by Tanimoto *et al* (2015), shows that the prevalence of CAS in CAD patients was between 7.0% to 36.0%. ^3^ Prevalence of AAA in CAD patients was studied by Durieux *et al* (2014), which shows 2.5% to 14.4% of total CAD cases had asymptomatic AAA. ^4^ The lower prevalence of PAD, CAS, and AAA in this sample study might be due to the younger mean age compared to studies conducted outside of Indonesia. It was known that the prevalence of PAD, CAS, and AAA increases with age. ^16^^–^^18^ In this study, the mean age of CAD patients was 60 years old. Patients with multi-vascular diseases were significantly older than patients without any vascular disease (64 vs 59; *p*<0.001). This was in line with another study involving CAD patients in Indonesia. A study conducted at Dr. Soetomo regional hospital in Indonesia by Saputri *et al* (2020) demonstrated the prevalence of CAD peaked in patients between 51–60 years old which consists of 42.6% of all CAD cases. The prevalence of CAD in patients older than 60 years old was only 33.1%. ^19^ Meanwhile, global data from 1990–2017 show that the peak prevalence of CAD happened in patients between 80 to 89 years old with a prevalence of 15,000–16,000 per 100,000 cases. ^20^ This shift of peak prevalence towards younger patients in Indonesia might be caused by the higher prevalence of CAD risk factors in the younger generation. This statement is supported by data from the study of Hussain *et al* (2016), which show higher population attributable-risk proportion (PAR) percentages of smoking, hypertension, excess body weight, diabetes, and hypercholesterolemia in CAD patients less than 55 years old compared to older patients. ^21^

Another factor that might influence the low prevalence of vascular disease in this study is the high rate of diabetes patients included. Our study data showed that 44.4% of patients had diabetes mellitus. Diabetes mellitus can decrease the rate of vascular disease in our study probably due to the protective effect of metformin medication on cardiovascular diseases. A randomized trial study called UK Prospective Diabetes Study (UKPDS) showed that in overweight patients newly diagnosed with diabetes mellitus type 2, metformin medication significantly decreases the relative risk of developing myocardial infarction, peripheral vascular disease, and microvascular disease. ^22^^,^^23^ In mouse and human macrophage studies, metformin was known to directly inhibit atherosclerosis through the activation of AMPK-ATF1-M2-like pathway and suppression of PI3K/AKT/mTOR/autophagy pathway, which was associated with the aneurysmal wall of the abdominal aorta. ^24^^,^^25^ Therefore the author concluded that the low prevalence of vascular disease especially AAA in this study might be caused by the high rate of diabetic patients included in this study.

There was a positive trend between the degree of CAD and the degree of atherosclerosis in another artery. The degree of fat deposit in coronary artery reflects the degree of atherosclerosis reflected on the increased serum level of interleukin-6 (IL-6) and leptin alongside the decreased serum level of adiponectin. ^26^ In this study, heightened coronary fat deposits in multivessel CAD patients was associated with atherosclerosis in other arteries, as indirectly demonstrated by an increased prevalence of multi-vascular disease. This trend was observed for the prevalence of multi-vascular disease, PAD, and CAS, while no significant difference was observed in the prevalence of AAA. This trend was also observed in another study. Kim *et al* (2013) conducted the study with the Korean population, where they observed a higher prevalence for bilateral PAD in multivessel CAD patients compared to patients with univessel CAD (5.3% vs 1.9%; *p*<0.001). Moreover, patients with multivessel CAD were at a higher risk for a more severe PAD, indicated by low ankle-brachial index (ABI) score (<0.7) compared to univessel CAD patients (6.3% vs 2.2%; *p*=0.008). ^27^ For carotid artery stenosis, Tanimoto *et al* (2005) reported an increase in prevalence of CAS in patients with zero, one, two, and three-vessel CAD with a prevalence of 7.0%, 14.5%, 21.4%, and 36.0% respectively ( *p*<0.0001). ^3^ This trend was also observed in AAA, where Durieux *et al* (2014) reported increasing prevalence of AAA in normal, one, two, and three-vessel CAD with a prevalence of 2.5%, 4.3%, 5.7%, and 14.4%, respectively. ^4^ Our data showed no significant increase of AAA prevalence in relation to different CAD severities. This might be caused by small prevalence of AAA found in this study, which influenced the estimation of statistical significance. This study offered a broader view of the relationship between vascular disease and multivessel CAD. To date, this is the only study that includes various vascular diseases in one population to see each diseases trend in prevalence between different severity of CAD.

This study shows several risk factors associated with increased vascular disease in CAD patients. Multivariate analysis showed that patients with CAD, ages of ≥60 years old, diabetes mellitus, cerebrovascular disease, and CAD3VD have higher odds of having multi-vascular disease. Both age and diabetes mellitus are known risk factors for atherosclerosis. Hisayama reported that age (hazard ratio (HR) 1.08; 95% CI 1.07-1.10; *p* <0.001) and diabetes mellitus (HR 1.58; 95% CI 1.17-2.14; *p*=0.003) increase the 10-year risk for atherosclerotic cardiovascular disease in the Japanese population. ^28^ The variable associated with the occurrence of another vascular disease somehow matched the factors associated with multivessel CAD. Predictive model made to predict high risk model based on data from PROMISE (Prospective Multicenter Imaging Study for Evaluation of Chest Pain) cohort shows that older age and diabetes mellitus increase the odds for multivessel CAD. ^29^ On the other hand, multivessel CAD could be a predictor for other vascular diseases. In a targeted screening study by Saw *et al* (2020), multivessel CAD had a higher prevalence of asymptomatic AAA when compared to univessel CAD, where patients with asymptomatic AAA also had CAD3VD. ^9^ Cerebrovascular disease, often manifested as stroke, is often a result of atherosclerosis. This study shows that the already manifested atherosclerosis in brain microvessel is a predictive factor for another atherosclerotic vascular disease in patients with CAD.

Analysis of risk factors significantly associated with the incidence of vascular disease in CAD patients resulted in a risk scoring system that enabled targeted screening. Risk scoring was made based from beta coefficient obtained from multivariate analysis. Goodness of fit test using Hosmer-Lemeshow test shows that the data fit the model. Predictive capability of risk score was shown to be moderately good with area under curve (AUC) of 65.9%. This predictive capability was comparable with another predictive model to predict PAD in patients with stable CAD made by Badheka *et al* (2011). In this study, ROC analysis of risk score that consists of history of hypertension, smoking, age, and history of diabetes showed AUC of 68.6%. ^30^ In our study, it was shown that general screening of all CAD patients resulted in only 15.4% cases with other vascular diseases and number needed to screen (NNS) was 6. Meanwhile, targeted screening for patients only with high-risk based on our risk score decrease the NNS from 6 to 3 with 45% of patients had any other vascular disease. Our clinical risk score shows a very high number of specificity of 96.5%. This high specificity means that this tool is less likely to produce a false positive result. Thus, it is more suited for ruling in the possibilities of multivascular disease instead of ruling out. We conclude that this tool is more suited for tertiary care hospital and should be applied to patients with doubtful Doppler result for screening of multivascular diseases.

### Study limitation

The main limitation of this study is the cross-sectional design, which does not study the potential for future vascular disease incidents and enabled bias. Potential bias in this study comes especially as a form of confirmatory bias by the assessor when diagnosing the incident of asymptomatic vascular disease. Although this confirmatory bias was minimized by involving third party examinators, this blinding was not properly controlled due to the number of patients involved in such short amount of time. Another limitation is the confirmation of the reduced NNS which used the same dataset with the one used to construct the risk scoring tool. External validation of our risk score using a different dataset is needed to confirm the generalizability of the study result.

## Conclusion

Patients who have diabetes mellitus, cerebrovascular disease, CAD3VD, and are above 60 years old are associated with increased odds of multi-vascular disease. By using risk scoring tool made from these risk factors, targeted screening in high-risk patients decrease the number needed to screen in half with high specificity.

## Author contributions

**Suko Adiarto**: Conceptualization, Methodology, Writing – Original Draft, Writing – Review & Editing, Data Curation, Visualization. **Luthfian Aby Nurachman**: Formal Analysis, Writing – Original Draft. **Raditya Dewangga:** Formal Analysis, Investigation, **Suci Indriani:** Investigation, Methodology **Taofan:** Investigation, Methodology **Amir Aziz Alkatiri:** Investigation, Methodology **Doni Firman:** Investigation, Methodology **Anwar Santoso:** Conceptualization, Validation, Writing – Review & Editing, Supervision, Funding Acquisition All authors read and approved the final paper.

## Data Availability

Figshare: Predicting Multi-Vascular Diseases_Dataset. DOI: http://doi.org/10.6084/m9.figshare.22881431. ^31^ This study contains the following underlying data: Predicting Multi-Vascular Diseases_Dataset.xlsx (data used for analysis). Data are available under the terms of the Creative Commons Attribution 4.0 International license (CC-BY 4.0).
