# Supplementary material for: Predicting multi-vascular diseases in patients with coronary artery disease
Source: F1000Res. 2023 Sep 12;12:750. Originally published 2023 Jun 26. [Version 2] doi: 10.12688/f1000research.134648.2 (PMC10517298; doi:10.12688/f1000research.134648.2)
Supplement: Peer review history

# Reviewer report: Reviewer response for version 2

**DOI:** 10.5256/f1000research.155734.r205642

**Published:** 2023-09-22

**Authors:** Marc Vuylsteke

**Affiliations:** 1 Saint Andries Hospital, Tielt, Flanders, Belgium

Thank you for the reply, no further questions

Is the work clearly and accurately presented and does it cite the current literature?

Yes

If applicable, is the statistical analysis and its interpretation appropriate?

Yes

Are all the source data underlying the results available to ensure full reproducibility?

Yes

Is the study design appropriate and is the work technically sound?

Yes

Are the conclusions drawn adequately supported by the results?

Yes

Are sufficient details of methods and analysis provided to allow replication by others?

Yes

Reviewer Expertise:

Vascular surgery

I confirm that I have read this submission and believe that I have an appropriate level of expertise to confirm that it is of an acceptable scientific standard.

# Reviewer report: Reviewer response for version 1

**DOI:** 10.5256/f1000research.147721.r182359

**Published:** 2023-07-14

**Authors:** Johannes Nugroho eko putranto

**Affiliations:** 1 Department Cardiology and Vascular Medicine, Faculty of Medicine, Airlangga University, Surabaya, East Java, Indonesia

The article presents a study aimed at constructing a predictive model and scoring system to enable targeted screening for multi-vascular diseases in patients with coronary artery disease (CAD). Overall, the article provides valuable insights into improving screening programs for CAD patients.

 The study methodology involves a cross-sectional design and the use of various diagnostic techniques. The inclusion and exclusion criteria are clearly defined, and the diagnostic techniques for various vascular diseases are explained. The article describes the statistical methods used, including univariate analysis, multivariable stepwise logistic regression, Hosmer-Lemeshow test, and the creation of a scoring system. The article also states that potential bias for each diagnosis of vascular disease was minimized by involving third-party examiners who were not aware of the existence of the study.

 The results demonstrate the effectiveness of the predictive model in reducing the number needed to screen (NNS) and achieving high specificity. The area under the curve (AUC) of the model is reported as 0.659, indicating good predictive ability. However, it would be useful to provide the sensitivity, specificity, positive predictive value (PPV), and negative predictive value (NPV) for the model at a chosen cutoff point. This information would further illustrate the diagnostic performance of the model. Overall, the discussion effectively acknowledges and discusses the study limitations. The discussion briefly mentions the need for external validation to confirm the generalizability of the risk scoring tool.

Is the work clearly and accurately presented and does it cite the current literature?

Yes

If applicable, is the statistical analysis and its interpretation appropriate?

Yes

Are all the source data underlying the results available to ensure full reproducibility?

Yes

Is the study design appropriate and is the work technically sound?

Yes

Are the conclusions drawn adequately supported by the results?

Yes

Are sufficient details of methods and analysis provided to allow replication by others?

Yes

Reviewer Expertise:

Cardiology and vascular medicine

I confirm that I have read this submission and believe that I have an appropriate level of expertise to confirm that it is of an acceptable scientific standard.

# Response

**Published:** 2023-09-09

**Authors:** Suko Adiarto

**Affiliations:** Dept. Cadiology and Vascular Medine, Universitas Indonesia/Harapan Kita NCVC Jakarta, Indonesia

Thank you for the suggestion. The sensitivity, specificity, positive predictive value (PPV), and negative predictive value (NPV) for the model at a chosen cutoff point were demonstrated in Table 5. Performance of risk score in detecting vascular disease in CAD patients. Because the cutoff point of our scoring system was two (three groups), we compared the at least moderate risk vs. low risk, and at least high risk vs. low risk.

# Reviewer report: Reviewer response for version 1

**DOI:** 10.5256/f1000research.147721.r182362

**Published:** 2023-07-10

**Authors:** Marc Vuylsteke

**Affiliations:** 1 Saint Andries Hospital, Tielt, Flanders, Belgium

This paper contains an interesting cross-sectional study regarding the risk factors of developping multi-vascular diseases in patients with coronary artery disease. However I do have some comments:

**Major comments**

1. Methods: Exclusion criteria: "We excluded patients with previously diagnosed CAD". But further you state: "Coronary artery lesions were considered as CAD if 2) the patient was previously hospitalized for treatment of coronary artery lesions". Isn’t that a contradiction?
2. Risk factors: 'hypertension': how do you quantify hypertension. Some patients have only very mild hypertension, some severe hypertension. Similar regarding PAD, diabetes. Is it possible to distinguish the severity of these conditions?
3. Risk-factors: ankle-brachial-index of >0.9: this is a very mild level of PAD. Most of those patients are asymptomatic and many of them will stay asymptomatic. Why not looking for the more severe form of PAD (ABI<0.4)? How do you assess ABI in patients with diabetes? As you know, this examination is not well reliable in diabetics given the presence of media sclerosis.
4. For assessing the risk of developing AAA, shouldn’t we look for a positive family history of AAA?
5. Table 1: Patient’s baseline characteristics: Metabolic syndrome was found in 51.1 % of included patients, however only 14.7% were obese? How can you explain this?
6. Please shorten the discussion significantly. The literature overview is much too long.

**Minor corrections**

1. Abstract, methods: 'coronary artery stenosis (CAS)', should be *Carotid artery stenosis.*
2. Introduction : line 3: 'carotid artery stenosis', please add 'CAS';
3. Methods: 'this study was conducted based on the STROBE guideline'. Please add reference.
4. Results: "A total of 1314 patients with CAD were identified; 203 patients have [multi vascular disease] *"*.

Is the work clearly and accurately presented and does it cite the current literature?

Yes

If applicable, is the statistical analysis and its interpretation appropriate?

Yes

Are all the source data underlying the results available to ensure full reproducibility?

Yes

Is the study design appropriate and is the work technically sound?

Yes

Are the conclusions drawn adequately supported by the results?

Yes

Are sufficient details of methods and analysis provided to allow replication by others?

Yes

Reviewer Expertise:

Vascular surgery

I confirm that I have read this submission and believe that I have an appropriate level of expertise to confirm that it is of an acceptable scientific standard, however I have significant reservations, as outlined above.

# Response

**Published:** 2023-09-08

**Authors:** Suko Adiarto

**Affiliations:** Dept. Cadiology and Vascular Medine, Universitas Indonesia/Harapan Kita NCVC Jakarta, Indonesia

Thank you very much for the review and the opportunity of revising the manuscript. Here is our comment by comment response:

**Major comments**

**1. Methods: Exclusion criteria: "We excluded patients with previously diagnosed CAD". But further you state: "Coronary artery lesions were considered as CAD if 2) the patient was previously hospitalized for treatment of coronary artery lesions". Isn’t that a contradiction?**

 We have revised this the sentence in the first paragraph of patient selection: *We excluded patients with previously diagnosed CAD that were treated by medical therapy and revascularization therapy* into *We excluded patients with previously diagnosed CAD that were hospitalized for other reason than elective coronary angiography or coronary angioplasty.*

**2. Risk factors: 'hypertension': how do you quantify hypertension? Some patients have only very mild hypertension, some severe hypertension. Similar regarding PAD, diabetes. Is it possible to distinguish the severity of these conditions?**

 In this study, we did not quantify the severity of hypertension, PAD and diabetes. We just simply categorized the patients whether they have hypertension, PAD or diabetes according to ESC guidelines, the value of ankle brachial index and ADA guidelines respectively.

**3. Risk-factors: ankle-brachial-index of >0.9: this is a very mild level of PAD. Most of those patients are asymptomatic and many of them will stay asymptomatic. Why not looking for the more severe form of PAD (ABI<0.4)? How do you assess ABI in patients with diabetes? As you know, this examination is not well reliable in diabetics given the presence of media sclerosis.**

 The purpose of this study is to screen the presence of multi-vascular disease in patients with proven coronary disease and subsequently propose a risk score that enable targeted screening. Thus, the screening would be for those who are considered high risk but asymptomatic.  Patients with ABI of <0.4 usually suffer from more severe PAD and would have already come for treatment. ABI is not a perfect diagnostic tool in the presence of media sclerosis; however it is still recommended for screening purposes by several professional organization through their guidelines (ESC, ACC/AHA, SVS, NICE) even in the presence of diabetes and chronic kidney disease.

**4. For assessing the risk of developing AAA, shouldn’t we look for a positive family history of AAA?**

 Very good point, unfortunately AAA is still underdiagnosed in our population. Thus the presence of family history might not be accurately identified.

**5. Table 1: Patient’s baseline characteristics: Metabolic syndrome was found in 51.1 % of included patients, however only 14.7% were obese? How can you explain this?**

 The criteria of metabolic syndrome was based on NCEP-ATP III Definition, in which 3 out of five criteria including waist circumference, blood pressure, LDL level, HDL Level and fasting glucose level. We re-analyzed metabolic syndrome using the definition, resulting in a prevalence of 37.7%. The difference between the 37.7% prevalence of metabolic syndrome and the 14.7% prevalence of obesity can be attributed to the presence of other criteria used to diagnose metabolic syndrome.

**6. Please shorten the discussion significantly. The literature overview is much too long.**

 We have shorten the discussion

**Minor corrections**

**1. Abstract, methods: 'coronary artery stenosis (CAS)', should be *Carotid artery stenosis.***

 Revised accordingly 

**2. Introduction : line 3: 'carotid artery stenosis', please add 'CAS'.**

 Revised accordingly

**3. Methods: 'this study was conducted based on the STROBE guideline'. Please add reference.**

 We have added the reference

**4. Results: "A total of 1314 patients with CAD were identified; 203 patients have [multi vascular disease] *"*.**

 Revised accordingly
